# Trends in Food Habits and Their Relation to Socioeconomic Status among Nordic Adolescents 2001/2002-2009/2010

**DOI:** 10.1371/journal.pone.0148541

**Published:** 2016-02-09

**Authors:** Anne-Siri Fismen, Otto Robert Frans Smith, Torbjørn Torsheim, Mette Rasmussen, Trine Pedersen Pagh, Lilly Augustine, Kristiina Ojala, Oddrun Samdal

**Affiliations:** 1 Department of Health Promotion and Development, University of Bergen, Bergen, Norway; 2 Division of Mental Health, Norwegian Institute of Public Health, Bergen, Norway; 3 Department of Psychosocial Science, University of Bergen, Bergen, Norway; 4 National Institute of Public Health, University of Southern Denmark, Copenhagen, Denmark; 5 School of Education and Environment, Kristianstad University, Kristianstad, Sweden; 6 Research Centre for Health Promotion, University of Jyväskylä, Jyväskylä, Finland; Chiba University Center for Forensic Mental Health, JAPAN

## Abstract

**Background:**

In the Nordic countries, substantial policy and intervention efforts have been made to increase adolescents' consumption of fruit and vegetables and to reduce their intake of sweets and soft drinks. Some initiatives have been formulated in a Nordic collaboration and implemented at national level. In recent years, social inequalities in food habits have been attracted particular governmental interest and several initiatives addressing the socioeconomic gradient in food habits have been highlighted. However, few internationally published studies have evaluated how trends in adolescents' food habits develop in the context of Nordic nutrition policy, or have compared differences between the Nordic countries.

**Methods:**

The study was based on Danish, Finnish, Norwegian and Swedish cross-sectional data from the international Health Behaviour in School-Aged Children (HBSC) study, collected via three nationally representative and comparable questionnaire surveys in 2001/2002, 2005/2006 and 2009/2010. Food habits were identified by students' consumption of fruit, vegetables, sweets and sugar sweetened soft drink. Socioeconomic status (SES) was measured with the Family Affluence Scale (FAS). Multilevel logistic regression was used to analyze the data.

**Results:**

Trends in fruit consumption developed differently across countries, characterized by an increase in Denmark and Norway and more stable trends in Sweden and Finland. Vegetable consumption increased particularly in Denmark and to a lesser extent in Norway, whereas Sweden and Finland displayed stable trends. Decreased trends were observed for sweet and soft drink consumption and were similar in Norway, Sweden and Finland. Sweet consumption decreased across all survey years, whereas soft drink consumption decreased between 2001/2002–2005/2006 and was stable thereafter. Denmark displayed an increase between 2001/2002–2005/2006 followed by a similar decrease between 2005/2006–2009/2010 for both sweet and soft drink consumption. Socioeconomic inequalities in fruit and vegetable consumption were observed in all countries, with no cross-country differences, and no changes over time. Small but not significant cross-country variation was identified for SES inequalities in sweet consumption. Reduced SES inequalities were observed in Sweden between 2005/2006 and 2009/2010. SES was not associated with soft drink consumption in this study population, with the exception of Denmark for the survey year 2009/2010.

**Conclusion:**

Different trends resulted in increased country differences in food habits during the time of observations. In survey year 2009/2010, Danish students reported a higher intake of fruit and vegetable consumption than their counterparts in the other Nordic countries. Finnish students reported the lowest frequency of sweets and soft drink consumption. Despite the positive dietary trends documented in the present study, the majority of Nordic adolescents are far from meeting national dietary recommendations. Our findings underline the need for more comprehensive initiatives targeting young people's food habits as well as a more deliberate and focused action to close gaps in social inequalities that affect food choices.

## Introduction

Improving young people’s food habits is of great importance in addressing overweight and chronic diseases [1–3]. This is relevant as food habits established in the early years tend to continue into adulthood [4–6]. Moreover, social inequalities in food habits are found to be less robust during teenage compared to other periods in life [7]. In the Nordic countries there is a governmental concern regarding adolescents not eating in accordance with national recommendations. As shown in international reports based on cross-sectional data from the Health Behaviour in School-aged Children (HBSC) collected in the time period 1997–2010 [8–11], many adolescents in Western countries do not include fruit in their everyday diet. Further, the overall prevalence of adolescents reporting daily intake of sugar-sweetened soft drinks is high. There is a socioeconomic dimension to this, as young people living in families with high socioeconomic status (SES) consume healthier diets than their counterparts living in families with low SES [12, 13, 10]. In order to improve this situation, substantial policy and intervention efforts have been implemented in the Nordic countries during the last decades with the aim of improving young people’s diet [14]. However, few internationally published studies have evaluated how adolescents’ eating habits develop in the context of Nordic nutrition policies.

Denmark, Finland, Norway and Sweden are all welfare states with a long tradition of governmental responsibility and Nordic collaboration on public health initiatives [15]. Cultural links between common lifestyle and dietary origins have provided a close collaboration between the countries, with common Nordic Nutrition Recommendations (NNR) [16], common action plans addressing public nutrition [17], collaborative research on “Nordic food” and development of coordinated monitoring systems among the pillars of their coordinated work. The common action plan includes a Nordic ambition that, by 2021, at least 70% of the population complies with the NNR recommendation of a daily intake of 500 grams of fruit and vegetables and that 80% meet the NNR recommendation for sugar consumption. Additionally, the Nordic ambition is to reach an SES variation of less than 20% in meeting the defined objectives with regard to diet, physical activity and overweight/obesity [17].

Adolescence is highlighted as a particularly important target group when aiming to reach the ambitions of improved dietary habits in the population as a whole and a variety of common structural and educational dietary incentives have been initiated in the Nordic collaboration and implemented at national level [18–23]. The context of national nutritional policies are previously described for Denmark [21] and Norway [19], in which different policy and educational incentives are discussed and the importance of the 5/6-a-day campaigns are emphasized.

Despite the close cooperation between the Nordic countries and common emphasise on young people’s diet, there are both similarities and differences in the specific choices of action taken by each government within the designated areas of common priority. As part of this, different ideologies across Nordic countries have resulted in different strategies for tackling social inequalities as well as diversities in how nutrition policies have been implemented [24]. One example concerning adolescents’ food habits in particular is the two different school lunch systems. As described by Kainulainen and colleagues [25], adolescents in Finland and Sweden are provided with hot school lunches consisting of meat/fish and vegetables, which is legally regulated and financially supported by national authorities. By contrast, adolescents in Norway and Denmark have a long tradition of bringing their own lunch boxes. Hot lunches are available for purchase from canteens in a limited number of schools [26]. However, in the time period 2007–2014 Norway provided all students in grades 8–10 with a free piece of fruit or carrot [27]. Additionally, several local initiatives [28–30] have been and are ongoing, aiming to improve food habits in the adolescent population.

Cross-national comparisons of Nordic dietary habits are of particular relevance for policy makers and others working in the field of public nutrition, because disparities in trends might reflect national differences in policies and efforts devoted to young people’s eating habits. Studies of developments over time are therefore relevant, although they do not allow for conclusions or assumption of causality. Further, analyses of trends in specified population groups may provide valuable knowledge on high-risk groups, e.g. low SES groups, as the influence of initiatives and programs may have differential effect on different SES groups. To our knowledge, no previous internationally published study has compared trends in young people’s food habits and their association with SES across Denmark, Finland, Norway and Sweden. The HBSC study [10] has provided brief data on how fruit and soft drink consumption differ in accordance with socioeconomic status for each survey year, but so far SES trends in nutritional behaviour have only been addressed using Norwegian [19, 31], Scottish [32] and Lithuanian data [33]. A recent publication presents trends across time from 33 HBSC-participating countries on fruit and vegetable consumption [34]. Trends from 2002–2010 are analysed for each individual country and increased consumption is found in most countries [34]. However, there is no statistical comparison of trends across countries. Using the same data set, the present study analyses trends in adolescents’ intake of fruit, vegetable, sweets and soft drinks, and their associations with socioeconomic status across Denmark, Finland, Norway and Sweden.

## Method

### The HBSC study

The present study is based on data from the HBSC study, which is a World Health Organization (WHO) collaborative cross-national survey with an overall aim to generate increased understanding of health and health behaviour, and their context in the lives of young people. The study was based on nationally representative samples of Danish, Finnish, Norwegian and Swedish students aged 15 participating in the HBSC study for the survey years 2001/2002, 2005/2006 and 2009/2010. Detailed information about the study is available at http://www.hbsc.org/

#### Sampling and response rates

The methodology for data collection is described in the HBSC protocol [35]. Samples were drawn by systematic cluster sampling using probability proportional to population size (PPS), thus yielding a nationally representative sample. The recommended national sample size was 1,500 and the mean age should be 15.5, where 90% of the sample should fall between +/- 6 months of the mean age. The students were recruited from schools (Denmark) or school classes (classroom unit) (Finland, Norway, Sweden). They completed an internationally standardized questionnaire at school after receiving instructions from their teacher. Students received oral and written information on the confidentiality of their responses. Participation was anonymous, voluntary and based on passive parental consent. The students were asked not to report their names on the questionnaire and to return the completed questionnaire in a provided sealed envelope, to ensure that only the researchers had access to this information. The international HBSC databank checked the quality of the data collected, performed appropriate cleaning of the data and merged national data sets into an international data file. Response rates for school, classes and students are shown in Table 1. Schools/classes that declined to participate, as well as students absent on the day the survey was carried out, were the two main sources of non-response and were not followed up. Further information concerning the sampling procedure is described by Roberts and colleagues [36].

**Table 1:** Response rate.

	Response rate (%) School/Classes/Student		
Survey year	2001/2002	2005/2006	2009/2010
Norway	73/88/89	69/69/84	55/56/81
Denmark	88/96/91	80/94/89	53/92/86
Finland	89/89/92	95/95/88	90/90/94
Sweden	84/84/87	83/83/85	88/88/77

#### Ethics

The HBSC study adheres to national regulations of research ethics and data protection and provides data that are comparable across countries and across time. Countries were required to follow the international research protocol [36], which prescribes consistency in sampling plans, survey instruments and data collection. Each participating country obtained approval to conduct the survey from the relevant ethics review board or equivalent regulatory institution, which is described below. In Denmark, there is no formal agency for ethical approval of school-based surveys. Approval for the study was requested through separate letters to the school board (parents' representatives), the school management and the schoolchildren's council in each of the participating schools. In Finland, ethical clearance of the study was approved by the Finnish Teachers' Union and the Finnish National Board of Education. Approval for the HBSC-study participation was requested through letters to the school principals. In Norway, the Privacy Ombudsman at the Norwegian Social Science Data Services confirmed that the study complied with privacy and confidentiality requirements. The study was sent to the Norwegian Western Regional Ethical Committee and was evaluated as not needing ethical clearance. In Sweden, surveys such as the HBSC-study falls under the Privacy act of The Swedish Data Protection Authority and ethical clearance is not needed. In all countries, the survey was based on passive consent, with the exception of a few Finnish schools where a notice of consent from parents was required

### Eating habits

The levels of consumption of fruit, vegetables, sweets and sugar sweetened soft drinks were measured by four questions on frequency of intake: “How many times a week do you consume fruit/vegetables/sweets/sugar sweetened soft drinks?” (Never = 0; Less than once a week = 1; Once a week = 2; Two to four times a week = 3; Five to six times a week = 4; Once a day = 5; More than once a day = 6). Each food frequency questions was dichotomized into 0 (no daily fruit/vegetables/sweets/soft drinks) response category 1–4) or 1 (daily fruit/vegetables/sweets/soft drink, response category 5–6).

One of the main aims of the HBSC study is to evaluate health behaviour and lifestyle patterns[10]. The outcome variables were therefore dichotomized to measure whether or not the students included fruit and vegetables, as well as sweets and soft drinks, in their everyday diet. This is in line with how these variables have been treated in other studies [12, 19, 37, 33] within the HBSC study.

### Socioeconomic status

#### The Family Affluence Scale (FAS)

The FAS is a measure of material affluence derived from the characteristics of the family’s household. FAS consists of the following four items: **1.** “Does your family own a car, van or truck?” (No = 0; One = 1; Two or more = 2). This item is a component of the Scottish Deprivation Index developed by Carstairs and Morris [38]. **2.** “How many times have you travelled away on holiday with your family during the past 12 months?” (Never = 0; Once = 1; Twice = 2; Three or more times = 3). This item is a measure of “deprivation of home facilities” [39]. **3.** “Do you have a bedroom to yourself?” (No = 0; Yes = 1). This item is a proxy for overcrowding, classified by Townsend [39] as housing deprivation, and is also a component of the Scottish Deprivation Index. **4.** “How many computers does your family own?” (None = 0; One = 1; Two = 2; More than two = 3). This item was introduced into the FAS to better differentiate between SES groups in affluent countries [40].

#### Ridit transformation of FAS

The individual FAS responses were combined and standardized by using ridit transformation to give a linear SES-score (0–1) within each country, having an overall mean score of transformed FAS = 0.5. The regression coefficient of the FAS score can be directly interpreted as the predicted difference in eating habits between the least deprived individual and the most deprived individual. When using this procedure, ordered categorical variables are converted to cumulative probabilities, and the individuals are thus ranked on this continuum. Ridit transformation is previously applied in inequality studies using SES scales with ordinal measurement [41–44] and recommended for comparisons of the effects of FAS [40].

### Statistical analysis

Gender adjusted frequencies of daily consumption of fruit, vegetables, sweets and soft drinks were calculated for each year of data collection. Time trends were identified by comparing survey years. Backward difference coding was used to compare 2005/2006 versus 2001/2002 and 2009/2010 versus 2005/2006. School class was included as a random effect. Intraclass correlations were derived from unconditional models and equalled .07, .08, .12 and .16 for daily fruit, vegetables, sweets and soft drink consumption respectively.

Time trends and trends in social inequality were analysed by means of multilevel mixed effects logistic regression for each country separately. Pooled analyses were performed to examine country differences, for which Norway was defined as the reference group.

The interaction effect was measured by Survey year * Time * Country and levels of significance is presented by p-values. All analyses were adjusted for gender.

The statistical analyses were performed in STATA version 13.1.

## Results

### Time trends in food habits

#### Fruit consumption

As shown in Table 2, a positive trend in adolescents’ fruit consumption was observed for Norway (OR 1.85, CI 1.54–2.23), Denmark (OR 1.66, CI 13.6–2.02) and Sweden (OR 1.36, CI 1.12–1.66) when comparing survey year 2001/2002 with 2005/2006. When comparing 2005/2006 with 2009/2010, a further increase in daily fruit consumption was observed in Denmark but not in any of the other Nordic countries. In Sweden, the proportion of students reporting daily fruit decreased (OR .83, CI .70-.99) during 2005/2006 to 2009/2010. In Finland, the proportions remained stable during the whole time period, demonstrated by no significant changes across the survey years. When comparing trends across countries, our analysis indicated that trends in Denmark developed similarly to Norway´s trends during 2001/2002 to 2005/2006 but not from 2005/2006 to 2009/2010, when Danish students reported a further increase in fruit consumption and stagnation was observed in Norway. Trends in Finland and Sweden were found to be significantly different from Norway during 2001/2002 to 2005/2006, but not from 2005/2006 to 2009/2010. In 2009/2010, the proportion of students eating fruit every day varied across countries. As shown in Table 2, relatively almost twice as many Danish students (45.2%) reported daily fruit than did Finnish (22.2%) and Swedish (24.2%) students in survey year 2009/2010.

**Table 2:** Proportion of and differences in daily food consumption by country and survey year, adjusted by gender.

	Proportion of students with daily consumption % (n)		
	2001/2002	2005/2006	2009/2010
**Fruit**			
**Norway**	24.1	36.3***	38.8
	(388)	(550)	(507)
Denmark	27.0	37.6***^a^	45.2**^a^
	(370)	(582)	(556)
Finland	20.2	20.9^b^	22.2^b^
	(350)	(358)	(473)
Sweden	22.1	27.9 **^b^	24.2*^a^
	(269)	(425)	(505)
Total	23.2	30.5**	30.3
	(1373)	(1913)	(2040)
**Vegetables**			
Norway	18.2	26.2***	28.4
	(293)	(397)	(373)
Denmark	24.7	35.4 ***^a^	41.1*^b^
	(336)	(547)	(504)
Finland	21.5	24.8^b^	24.8^a^
	(374)	(422)	(530)
Sweden	29.8	34.1^a^	34.5 ^a^
	(362)	(517)	(711)
Total	23.0	30.0**	31.4
	(1364)	(1880)	(2112)
**Sweets**			
Norway	18.9	13.0***	9.0**
	(304)	(199)	(120)
Denmark	11.3	14.1*^b^	10.0**^a^
	(153)	(218)	(121)
Finland	9.1	6.1**^a^	3.9**^a^
	(159)	(102)	(83)
Sweden	15.2	9.6**^a^	8.9**^a^
	(185)	(145)	(185)
Total	13.5	10.6**	7.6**
	(801)	(665)	(508)
**Soft drinks**			
Norway	27.1	17.4***	14.7
	(436)	(227)	(199)
Denmark	11.2	13.9*^b^	11.0*^a^
	(149)	(215)	(131)
Finland	8.3	6.2 *^a^	5.0^a^
	(145)	(101)	(105)
Sweden	14.3	9.7**^a^	8.9^a^
	(175)	(146)	(185)
Total	15.3	11.7**	9.2**
	(906)	(732)	(619)

a = trend is not significantly (p>.05) different from Norway.

b = trend is significantly (p < .05) different from the Norway.

***/**/* significant difference from last survey year p < .001/.01/.0

#### Vegetable consumption

As shown in Table 2, a positive trend in adolescents’ vegetable consumption was observed for Norway (OR 1.62, CI 1.33–1.97) and Denmark (OR 1.74, CI 1.41–2.14) when comparing survey year 2001/2002 with 2005/2006. A further increase in the proportion of students eating vegetables every day was documented in Denmark when comparing 2005/2006 with 2009/2010 (OR 1.27, CI 1.03–1.55) but not for Norway. In Finland and Sweden, no significant changes in the proportion of students reporting daily vegetables were found across the survey years. When looking at overall country differences, our analyses indicated that trends in vegetable consumption in Denmark, Finland and Sweden developed similarly to the Norwegian trend. However, in the time period 2005/2006 to 2009/2010, vegetable consumption showed a greater increase in Denmark than Norway. As shown in Table 2, Denmark had the highest proportion of students reporting daily vegetable consumption in survey year 2009/2010 (41.1%), followed by Sweden (34.5%), Norway (28.4%) and Finland (24.8%).

#### Sweets consumption

As shown in Table 2, a reduced proportion of students reporting daily sweet consumption was found in Norway (OR .64, CI .51-.88), Finland (OR .62, CI .46-.85) and Sweden (OR .59, CI .42 - .84) when comparing survey year 2001/2002 with 2005/2006. By contrast, increased sweet consumption was identified in Denmark (OR1.31, CI 1.01–1.70). When comparing 2005/2006with 2009/2010, a further significant decrease in sweet consumption was also found in Norway (OR .67, CI .51-.88) and Finland (OR .63, CI .45-.88) but not in Sweden. During this time period, decreased sweet consumption was identified in Denmark (OR .66, CI .50-.87). When analysing country differences the trends in sweet consumption in Finland and Sweden did not differ significantly from the Norwegian trend during the whole period of observations. Trends in Denmark differed from the Norwegian during 2001/2002 to 2005/2006, characterized by increased sweet consumption.

#### Soft drink consumption

As shown in Table 2, the proportion of students reporting the consumption of soft drinks every day decreased in Norway (OR .56, CI .45-.70), Finland (OR .72, CI .52–1.00) and Sweden (OR .62, CI .44-.88) when comparing survey year 2001/2002 to 2005/2006. No statistically significant differences were found for all three countries when comparing 2005/2006 with 2009/2010. By contrast, increased soft drink consumption was observed in the Danish sample during 2002 with 2006 (OR 1.32, CI 1.00–1.73). In the time period 2006 to 2010, the proportion decreased (OR .74, CI .56 - .97). When analysing country differences, our analysis indicated that trends in soft drink consumption developed similarly in the Nordic countries, except for the Danish trend in the period 2001/2002 to 2005/2006. This was significantly different from the Norwegian trends.

### Social inequality in food habits

#### Fruit consumption

As shown in Table 3, social inequality in fruit consumption was documented in Norway in 2005/2006, in Denmark in 2005/2006 and 2009/2010 and in all survey years in Finland and Sweden. In all strata students with a higher FAS score were more likely to report daily fruit consumption. Our analysis indicated that the trends in social inequality in fruit consumption did not differ across countries (p = .62). Moreover, social inequality remained stable across survey years (p = .62).

**Table 3:** OR (95% CI) for the association between family affluence and daily consumptions by country and survey year, adjusted for gender.

	2001/2002	2005/2006	2009/2010
	OR (95% CI)	OR (95% CI)	OR (95% CI)
**Fruit**			
Norway	1.55 (1.00–2.42)	1.64 (1.10–2.45)	1.40 (.89–2.21)
Denmark	1.50 (.96–2.35)	1.61^a^ (1.07–2.42)	2.13^a^ (1.40–3.38)
Finland	2.32 (1.48–3.64)	2.38^a^ (1.51–3.78)	1.75^a^ (1.18–2.57)
Sweden	1.27 (.78–2.07)	1.60^a^ (1.06–2.41)	1.81^a^ (1.24–2.65)
Total	1.64 (1.31–2.05)	2.02 (1.64–2.49)	2.13 (1.73–2.62)
**Vegetable**			
Norway	1.68 (1.03–2.73)	2.36 (1.50–3.70)	1.89 (1.16–3.10)
Denmark	1.97 (1.24–3.12)	1.50^a^ (.99–2.26)	3.09*^a^ (1.95,-4.89)
Finland	2.24 (1.42–3.54)	2.08^a^ (1.33–3.23)	1.82^a^ (1.23–2.68)
Sweden	1.64 (1.04–2.59)	1.53^a^ (1.01–2.30)	1.95^a^ (1.37–2.77)
Total	1.75 (1.39–2.19)	1.79 (1.45–2.20)	2.11 (1.73–2.59)
**Sweets**			
Norway	1.71 (1.06–2.76)	.81 (.46–1.43)	.78 (.37–1.61)
Denmark	.61 (.32–1.16)	1.03^a^ (.60–1.77)	.56^a^ (.28–1.14)
Finland	1.58 (.85–2.94)	.1.02^a^ (.47 2.21)	.54^a^ (.23–1.30)
Sweden	.79 (.44–1.42)	1.54^a^ (.79–3.02)	.47**^a^ (.26-.85)
Total	1.37 (1.03–1.81)	1.25 (.92–1.70)	.72* (.51–1.01)
**Soft drink**			
Norway	1.04 (.68–1.58)	.94 (.57–1.58)	.77 (.42–1.39)
Denmark	.63 (.33–1.20)	.65 (.37–1.15)	.47 (.23-.95)
Finland	.89 (.46–1.72)	1.20 (.55–2.61)	1.26 (.59–2.69)
Sweden	1.26 (.68–2.33)	1.07 (.55–2.09)	.54 (.30-.97)
Total	1.25 (.95–1.66)	1.15 (.85–1.56)	.92 (.67–1.27)

OR = Odds ratio, CI = Confidence interval.

a = trend is not significantly (p>.05) different from Norway.

**/* significant difference from last survey p < .01/.05

#### Vegetable consumption

As shown in Table 3, a social gradient in vegetable consumption, that is students with higher FAS being more likely to report eating vegetables every day, was found in all countries for every year of data collection, except for the Danish sample in the survey year 2005/2006. Trends in social inequality in vegetable consumption did not differ across countries (p = .34) and remained stable during the time period 2001/2002 to 2009/2010 (p = .55).

#### Sweet consumption

Except for Norway in survey year 2001/2002 (OR 1.71, CI 1.06–2.76) and Sweden in survey year 2009/2010. (OR .47, CI .26-.85), no significant association between SES and sweet consumption was found among Nordic adolescents. The trend in the social gradient of sweet consumption did not differ across countries (p = .16). A reduction of social inequality in sweet consumption was observed over time (p = .01), more specifically between 2005/2006 and 2009/2010 (OR .55, CI .34-.87).

#### Soft drink consumption

Except for Denmark (OR .47, CI .23-.95) in survey year 2009/2010, no significant association was found between soft drink consumption and SES. The four countries followed similar trends (p = .74) with no changes across time (p = .25).

## Discussion

During the time period 2001/2002 to 2009/2010 there was an increase in daily fruit and vegetable intake in Norway and especially in Denmark, while the overall numbers in Finland and Sweden were quite stable. While students in Denmark increased their intake of fruit and vegetables, they did not reduce their intake of sweets and soft drinks and this consumption remained quite stable. In Finland, Norway and Sweden, consumption of sweets and soft drinks decreased during the time of observations. Finland and Sweden show similar trends in eating habits but at different levels. The greatest changes across survey years were identified in the time period from 2001/2002 to 2005/2006. The overall results regarding social inequalities in eating habits identified socioeconomic inequalities across all countries and survey years with regard to fruit, vegetables and sweets, but not soft drinks. Few trend differences were observed between countries. Reduced socioeconomic differences in consumption of sweets were observed in Sweden during 2005/2006–2009/2010. No inequalities were observed in soft drink consumption, with the exception of Denmark for the survey year 2009/2010.

Our findings are in line with previous national studies documenting increased fruit and vegetable consumption in Scotland (2002–2010) [32], the Netherlands (2003–2009) [45] Norway 2001–2008 [31] and among Lithuanian girls (2002–2010) [33]. In the HBSC publication by Vereecken and colleagues [34], increased fruit consumption was documented for two-thirds, and vegetable consumption for half, of the 33 countries during 2002 to 2010, including Norway and Denmark. In contrast to the present study, Vereecken and colleagues found increased fruit consumption in Finland, and increased vegetable consumption in Sweden. However, Vereecken and colleagues [34] included data from students aged 11, 13 and 15 year, while the present analysis is based on 15-year-olds only. This may explain the differences between our findings and the findings by Vereecken and colleagues. The findings of decreased soft drink consumption are in line with studies conducted in The Netherlands (2003–2009) [45], USA (1999–2010) [46], in Lithuania 2006–2010 [33] and also in a previous Norwegian study (2001–2008) [47]. Stable socioeconomic differences in adolescents’ food habits are documented in Scotland (2002–2010) [32] and Lithuania [33] as well as among Danish adults [48]. The majority of the studies [32, 19, 21, 33] referred to above are based on data from the HBSC study, which make comparisons across countries particularly relevant due to common protocol for data collection. The study extends previous work by indicating that high SES groups are more likely than low SES groups to respond to national dietary recommendations [7, 49].

The present study has a descriptive focus and does not evaluate nutrition policies in the respective countries. However, it identifies how trends developed in the current context of Nordic nutrition policies, indicating the relevance of interpreting the findings in light of the recent decade’s initiatives addressing adolescent nutrition [50, 51, 28, 30, 21, 19]. Free school fruit and fruit subscription programmes (parental payment) are examples of initiatives that have been shown to increases adolescents’ fruit intake [27, 50, 30]. Although the extension of such initiatives cannot be estimated, the effect may vary by country and contribute to understanding the observed differences between the Nordic countries. Improved dietary habits are also found in the adult populations [52, 53], indicating improved food habits as a general societal trend in the Nordic countries and thereby further underlining the possible positive impact of the Nordic nutrition policies and initiatives on individual level behaviours.

The observed trends in fruit and vegetable consumption demonstrate increased differences across the Nordic countries during the years 2001/2002 to 2009/2010. In 2009/2010, approximately twice as many Danish (45%) as Finnish (22%) and Swedish (24%) students reported daily fruit consumption. This leaves Danish adolescents as the highest—and Finnish adolescents as the lowest—fruit consumers, not only among the Nordic countries but also among adolescents in other European [54, 34] countries and the USA [34]. The lowest number of both daily sweets and soft drinks consumers was identified in the Finnish sample. It is remarkable that despite the relatively low number of daily consumers observed in Finland in 2002, reduced sugar consumption was observed through the whole time period. The present analysis indicates that social inequalities in food habits remained relatively stable through the time of observations, which might suggest that the Nordic governments have not succeeded in reducing dietary inequalities. On the other hand, initiatives such as mass media strategies, which have been widely used to communicate the 5/6-a-day campaigns, are suggested to widen dietary inequalities by favouring high SES groups in their communication strategy [55, 56]. The overall indication of unchanged dietary inequalities in the adolescent population may indicate that the 5/6-a-day-campaigns have reached all SES groups and are therefore worth encouraging.

### Strengths and limitations

As far as we know, the present study is the first to analyse nationally representative data on adolescents’ food habits in a comparative Nordic perspective and is of particular relevance in the field of public nutrition in the Nordic countries. The study is moreover unique in an international perspective because comparable data allows further and future comparison with adolescents’ eating behaviours in other European countries participating in the HBSC survey as well as in the USA and Canada, participating in the HBSC survey.

The study has limitations that should be considered when interpreting the findings. The purpose of the HBSC study is research on health and health behaviours and the HBSC questionnaire only assesses frequency of consumption and not the amount of fruit, vegetable, sweets and soft drinks consumed by the students. We can therefore not conclude whether the total consumption of the respective food items has changed during 2001/2002 to 2009/2010. However, the questionnaire is recognized as a valid instrument in epidemiological studies ranking adolescents according to their usual food intake [57] and is therefore considered valid for measuring whether adolescents include fruit, vegetables, sweets and soft drinks in their everyday diet.

When exploring social inequalities, the use of SES indicators should always be taken into consideration. In the present study SES was measured by FAS, which is a material indicator that was developed to measure material wealth among young people in Europe and North America. As shown by Schnohr and colleagues [40], the reliability of FAS varies between countries and is found to be lower in the Nordic countries compared with other European countries. However, ridit transformation of FAS, thus using relative rather than absolute score, compensates for some of the limitations and makes the FAS a relevant indicator of material wealth in Nordic conditions. Ridit transformation is indeed recommended when measuring FAS variation across countries [40].

We underscore that the association between SES and food habits could be different if FAS was replaced with an indicator tapping educational or cultural dimensions of the SES construct. Social inequalities are documented in soft drink consumption among adolescents when education [13, 58, 59] and cultural capital [12] are used as an SES indicator. This demonstrates the challenges in social inequality research among young people.

### Implications for practice

Although the present study indicates increased fruit and vegetable consumption in Denmark and Norway, the majority of Nordic adolescents do not respond to national recommendations of including fruit and vegetables in their everyday diet. A specific challenge is to increase vegetable intake and new approaches might be needed to capture young people’s interest in such food items. Fruit and vegetables are found to be more strongly associated with SES than are other food items [60–62], which indicate that initiatives to reduce social inequalities in fruit and vegetable consumption should be explored and prioritized. Building on the principles of Bronfenbrenner’s Ecological System Theory (EST)[63], food habits are shaped through the individual’s interaction with environmental determinants. School and leisure time activities are arenas that should be targeted more systematically. Importantly, they represent adolescents’ everyday context and reach adolescents across different SES groups.

Initiatives addressing the high consumption of sweets and soft drinks should also be emphasized. The observed decrease in soft drink consumption is encouraging, but the need for further decrease is urgent, particularly among Norwegian students who were identified as the highest number of soft drink consumers for every survey year of data collection. The Danish government should notice that, while daily sugar intake decreased steadily in Norway, Finland and Sweden, this was not the case in Denmark. The Danish government increased the taxes on sugar products like sweets and soft drinks in 2010. This initiative and should be evaluated because similar incentives are currently debated also in the other Nordic countries.

### Implications for research

Future research should emphasize the need for more systematic evaluation of the effectiveness of policies and initiatives aiming to improve young people’s food habits. In line with Bronfenbrenner’s EST[63], this will imply defining, collecting and categorizing characteristics from different socio-ecological perspectives, e.g. national policies, nutritional recommendations and school based initiatives. Such studies should be done not only at national level but also in a comparative perspective, because structural differences between countries, such as different lunch models, may influence the effect of nutrition policy initiatives on individual level behaviours. In addition, the use of social media, to spread knowledge and healthy recipes, and to make healthy food trendy, should be evaluated. Such initiatives may be of particular importance when targeting adolescents as most teenagers sign up to e.g. Facebook and Twitter, which are currently used by the governments to spread information about nutrition and physical activity. Moreover, as a basis for more efficient differentiation of interventions to comply with specific characteristics of different target groups, future research should evaluate which factors contribute to social inequalities in food habits, as well as elaborate our knowledge of which health promotion strategies are the most effective in high-risk population groups.

Finally, there is also a need for monitoring adolescent food habits in the future. Data from the HBSC 2013/2014 survey will be published in the international report in 2016 and will be subsequently available for further investigation. Although our study indicates that dietary inequalities remained stable, socioeconomic differences should be followed carefully as they are shown to be widening in other types of adolescent health behaviour and in population-based health, including overweight/obesity among Swedish [64] and Danish [65] children and physical activity among Finnish adolescents [66].

## Conclusion

During the time period 2001/2002 to 2005/2006, the proportion of students including fruit in their everyday diet showed grater increase in Denmark and Norway compared with Finland and Sweden. Decreased sugar intake was documented in Finland, Norway and Sweden but not in Denmark. Different trends resulted in increased country differences in daily fruit/vegetable consumption during the time of observations. However, despite the positive dietary trends documented in the present study, the majority of Nordic adolescents are far from meeting the recommendation. Furthermore, clear and persistent patterns of social inequality remain. Our findings underline the need for more comprehensive initiatives targeting young people’s food habits. These should be based on systematic evaluations of public health initiatives addressing adolescents’ food habits. Finally, further monitoring of socioeconomic patterns in adolescents’ food habits should be highlighted and considered in the planning of future health promotion targeting adolescent nutrition.

## Abbreviations

FAS: Family affluence scale
HBSC: Health Behaviour in School-Aged Children
OR: Odds ratio
CI: Confidence interval
SES: Socio-economic status
WHO: World Health Organization
